# Epidemiological investigation and clinical characteristics study of orthopedic infections caused by *Aeromonas* in the southeast coastal region from 2018 to 2024

**DOI:** 10.1186/s12879-025-12141-5

**Published:** 2025-11-26

**Authors:** Liang Guo, Chaoyu Zhang, Xuejun Wu, Peisheng Chen, Zhiwei Wang, Yuning Li, Jin Yu, Fengfei Lin

**Affiliations:** 1 School of Clinical Medicine, Fujian Medical University, Fuzhou, Fujian 350122 China; 2 Department of Hand and Foot Microsurgery, Fuzhou Second General Hospital, Fuzhou, Fujian 350007 China; 3 Department of Plastic and Aesthetic Surgery, Fuzhou Second General Hospital, Fuzhou, Fujian 350007 China; 4 Department of Orthopedics, Fuzhou Second General Hospital, Fuzhou, Fujian 350007 China

**Keywords:** *Aeromonas* infection, Epidemiology, Clinical characteristics, Antibiotic resistance analysis, Logistic regression modelling

## Abstract

**Objective:**

To investigate the clinical and epidemiological characteristics of trauma-related orthopedic *Aeromonas* infections and analyze prognostic risk factors to guide early diagnosis and inform clinical management.

**Methods:**

This retrospective study analyzed 111 patients with traumatic *Aeromonas* infection from May 2018 to May 2024. Data on demographics, injury characteristics, laboratory parameters, treatment details, and outcomes were collected. Independent predictors of unfavorable prognosis were identified through univariate and multivariate logistic regression, with model performance evaluated via likelihood ratio test, ROC analysis, Hosmer-Lemeshow test, and confusion matrix.

**Results:**

A total of 111 patients with trauma-related *Aeromonas* infection were collected, with an average age of 49.9 ± 14.3 years. The average length of hospital stay was 36.2 ± 21.1 days, and the average number of surgeries was 2.7 ± 1.6. The outcomes included debridement in 51(45.95%) cases, skin grafting in 17(15.32%) cases, flaps in 27(24.32%) cases, amputation in 14(12.61%) cases, and death in 2(1.80%) cases. The drug sensitivity test results showed that *Aeromonas* extracted from the wound secretions were resistant to ampicillin and amoxicillin and were sensitive to amikacin, levofloxacin and cefepime. Our research has listed three predictive indicators significantly associated with unfavourable prognosis, mainly including body mass index (BMI), skin defect and perioperative blood transfusion.

**Conclusion:**

This study outlines the clinical profile of traumatic *Aeromonas* infection, which predominantly affects middle-aged individuals and is associated with extended hospital stays, frequent surgical interventions, and significant rates of adverse outcomes, including amputation (12.61%) and mortality (1.80%). Multivariate analysis identified BMI > 23.7 kg/m², skin defects, and perioperative blood transfusion as independent predictors of poor prognosis. For high-risk trauma patients with these factors, our model recommends prophylactic use of third-generation cephalosporins (e.g., ceftazidime), aminoglycosides (e.g., amikacin), or combination therapy in place of first-line cephalosporins. Vigilant perioperative monitoring for invasive infections is crucial, especially in immunocompromised patients.

**Clinical trial number:**

Not applicable.

## Introduction

*Aeromonas* is a type of Gram-negative facultative anaerobic bacillus, with a relatively high content in water bodies during warm seasons [1]. As a pathogen of zoonotic diseases, it has strong pathogenicity to both aquatic animals and humans. *Aeromonas* species have been identified as the etiological agent of diarrhea in approximately 2% of travelers to Africa, Latin America, and Asia [2]. In recent years, with the development of aquaculture and the aggravation of environmental water pollution, the number of *Aeromonas* infection cases has shown a significant upward trend. The global prevalence of *Aeromonas*-carried MCR-3, a key polymyxin resistance gene, has risen between 1968 and 2022. This trend directly threatens the efficacy of polymyxin B and E, vital last-line defenses against multidrug-resistant Gram-negative bacteria [3]. The World Health Organization has listed it as one of the emerging waterborne pathogens [4], warranting greater attention and vigilance.

In clinical practice, *Aeromonas* infection caused by contact with contaminated water sources after orthopedic trauma has become a severe challenge for surgeons [5]. Such infections often progress rapidly and can develop from local infection to necrotising fasciitis (NF), sepsis or even multiple organ failure within 48 h, posing a great threat to the patient’s life. Studies have found that *Aeromonas hydrophila (A. hydrophila)* infection significantly increases the risk of NF, a potentially life-threatening complication, with a reported mortality rate of 29.4% [6, 7]. Given the high mortality rate and rapid progression characteristics of traumatic *Aeromonas* infection in the extremities, establishing a reliable prognostic risk prediction model has critical clinical value.

Taking the prediction of drug resistance models as an example, in response to the problem of insufficient accuracy in predicting the sensitivity and drug resistance of *Pseudomonas aeruginosa (P. aeruginosa)* in global large-scale genomic databases, Danielle E. Madden et al. integrated a large database of specific antimicrobial resistance variations of *P. aeruginosa* and combined it with ARDaP software. It can predict the sensitivity/mediator/drug resistance of *P. aeruginosa* against 10 commonly used clinical antibiotics with a relatively high accuracy rate [8]. In the context of infectious diseases, the drug resistance prediction model can be regarded as a submodule of the disease prediction model. Drug resistance information directly influences treatment decisions, thereby affecting disease prognosis. Both models rely on similar types of data, such as clinical features and laboratory results. A scoring system may integrate drug resistance prediction results as one of the risk factors, thereby enhancing the accuracy of disease prediction.

## Materials and methods

### Subjects

A retrospective analysis was conducted on patients with traumatic *Aeromonas* infection of the orthopedics admitted to the Second General Hospital of Fuzhou from May 2018 to May 2024. Routine pathogen testing, which included screening for *Aeromonas*, was performed for all patients with open injuries, as well as those with closed injuries who presented with wound secretions [9]. During the study period, a total of 45,221 trauma patients were screened, and 136 cases of *Aeromonas* infection were identified, representing an overall incidence rate of 0.3%. Based on the Inclusion and Exclusion Criteria, 25 cases were excluded, resulting in a final cohort of 111 enrolled patients. General information, injury scenes, clinical manifestations, laboratory indicators, treatment process indicators, and prognostic indicators were collected.

#### Inclusion and exclusion criteria

Inclusion Criteria: This study enrolled patients of all ages who were admitted for orthopedic trauma and were diagnosed with an *Aeromonas* infection through pathogen detection.

Exclusion Criteria: Exclusion criteria comprised patients with chronic wounds, immunosuppression, significant comorbidities, as well as those who developed co-infections during the treatment course.

#### Diagnostic criteria

(1) Clinical presentation: Infections by *Aeromonas* typically manifest as skin and soft tissue infections. Inadequate or delayed therapeutic intervention, the infection may rapidly progress to high fever, chills, and invasive infections, including NF, myositis, and osteomyelitis, especially in immunocompromised patients [10, 11].

(2) Laboratory examination: Initial identification of *Aeromonas* species was based on Gram-negative rod morphology and a positive oxidase test. Final confirmation was obtained through culture of wound exudate and automated system analysis.

#### The definition of prognosis

The patients were divided into a favourable prognosis group and an unfavourable prognosis group based on the clinical outcomes. *Aeromonas* infections frequently lead to extensive superficial venous thrombosis, fascial and skin necrosis. An increase in the total number of surgeries and debridement procedures often suggests a high bacterial load or recurrent infection, which is a key contributor to an unfavorable patient prognosis [12, 13]. Given the lack of unified standard for unfavourable prognosis in clinical practice, we define poor prognosis as including the following situations: with death or transfer to another hospital as the outcome; For patients with amputation, the number of surgeries exceeds one; For patients undergoing flap treatment, the number of operations exceeds two; For patients with skin grafts, the number of surgeries exceeds four. Notably, the number of surgeries related to poor prognosis refers to the number of surgeries during the first hospitalisation after trauma, mainly including debridement, skin grafting, flap repair and amputation.

### Methods

#### Specimen collection

No antibiotics had been administered to any patient prior to sample collection. In the operating room, the wound surface was cleansed using sterile 0.9% saline irrigation. Subsequently, 3–5 secretion samples were collected from the wound with sterile cotton swabs. The swabs were immediately transferred into pre-labeled sterile containers containing transport medium, and the caps were securely fastened to avoid contamination.

#### Bacterial isolation and identification

Specimen collection and bacterial isolation were performed according to the “National Clinical Laboratory Operating Procedures” (4th edition), and the bacteria were isolated from cultures of wound secretions. The bacterial strains were identified using the VITEK-2 fully automated microbial analysis system (bioMérieux, France) and Microflex LT/SH Automated Microbial Identification System (Bruker Daltonics, Germany).

#### Antibiotic susceptibility testing

Susceptibility testing used disk diffusion and broth microdilution methods on 17 antibiotics: ampicillin, cefazolin, amoxicillin-clavulanic acid (disk diffusion), tetracycline (disk diffusion), trimethoprim-sulfamethoxazole, chloramphenicol (disk diffusion), cefotaxime (disk diffusion), ciprofloxacin, ceftazidime, levofloxacin, gentamicin, piperacillin-tazobactam, imipenem, aztreonam, cefepime, amikacin, and meropenem. Unspecified antibiotics were tested by broth microdilution. The results were interpreted by the guidelines of the Clinical and Laboratory Standards Institute (CLSI) [14]. The quality control strain used was *Pseudomonas aeruginosa* (ATCC27853), provided by the Ministry of Health Clinical Laboratory Centre.

#### Logistic regression modelling

Based on univariate analysis, variables associated with the outcome were initially screened. A logistic regression model was developed with prognosis type as the dependent variable (good = 0, poor = 1) and factors such as age as independent variables. Variable importance was assessed using odds ratios derived from the regression coefficients. The model’s overall validity was evaluated with the likelihood ratio test, its discriminative ability via area under the ROC curve (AUC), and its classification performance based on a confusion matrix reporting accuracy, sensitivity, and specificity.

### Statistical analysis

The data were analysed using SPSS 25.0 software. Measurement data were analysed using the t-test or the Mann-Whitney U test. The data on counting data were analysed using the chi-square test or Fisher’s exact test. The Spearman rank correlation coefficient analysis was used to compare the strength of the relationship between the two variables. For multiple groups of ordered categorical variables, the distribution was compared using Ridit analysis. Multivariate analysis was performed using a binary logistic regression model, with the test level α = 0.05. The model’s overall validity was assessed using the likelihood ratio test, while its discriminatory ability was evaluated by ROC curve analysis. Calibration and prediction accuracy were examined using the Hosmer-Lemeshow test and a confusion matrix, respectively. It is considered that *p* < 0.05 has a significant statistical difference.

## Results

### General characteristics of included patients

Over the 6-year period, 111 cases of *Aeromonas* infection were detected. Generally, males comprised 77.5% of all patients, compared to 22.5% females. Although a higher proportion of female patients had favorable outcomes (72% vs. 55.8% in males), Pearson’s chi-square test showed no statistical significance (*p* = 0.147). Despite the lack of statistical significance, the observed difference suggests that gender may be an important prognostic factor, with females potentially having better outcomes than males.

The BMI fluctuated between 18.4 and 39.2, with an average of 25.0 ± 4.0. 74.8% of the patients were within the normal and overweight range (BMI ranging from 18.5 to 27.9), among whom the proportion of those with an unpromising prognosis was 33.7%. Among the obese patients (BMI > 28), the proportion of those with poor prognosis was as high as 65.4% (Table 1), indicating that body weight may be an influencing factor of guarded prognosis.

**Table 1:** Demographic and clinical characteristics of patients with *Aeromonas* infection

Variable	Parameter	Prognosis		n	X²	*p*-value
		Favorable	Unfavourable			
Age	[9,27)	6(85.71%)	1(14.29%)	7	-	0.190
	[28,46)	16(50.00%)	16(50.00%)	32		
	[47,65)	34(57.63%)	25(42.37%)	59		
	[66,84]	10(76.92%)	3(23.08%)	13		
Gender	Female	18(72.00%)	7(28.00%)	25	2.105	0.147
	Male	48(55.81%)	38(44.19%)	86		
BMI	Underweight	2(100.00%)	0(0.00%)	2	9.616	0.008**
	Normal-overweight	55(66.30%)	28(33.70%)	83		
	Obesity	9(34.60%)	17(65.40%)	26		
Injured part	Upper arm	3(50.00%)	3(50.00%)	6	6.051	0.534
	Forearm	5(71.43%)	2(28.57%)	7		
	Hand	8(50.00%)	8(50.00%)	16		
	Thigh	4(100.00%)	0(0.00%)	4		
	Cnemis	23(54.76%)	19(45.24%)	42		
	Foot	11(57.89%)	8(42.11%)	19		
	Multiple parts	10(66.67%)	5(33.33%)	15		
	Other	2(100.00%)	0(0.00%)	2		

Note: ***, **, and * represent significance levels of 5%, 1% and 0.1% respectively

### Clinical characteristics of included patients

A total of 111 patients with trauma-related *Aeromonas* infection, with orthopedic trauma, were collected from 2018 to 2024. The outcomes included debridement in 51 cases(45.95%), skin grafting in 17(15.32%) cases, flaps in 27(24.32%) cases, amputation in 14(12.61%) cases, and death in 2(1.80%) cases. The number of surgeries ranged from 0 to 7 times, with an average of 2.7 ± 1.6 times and a median of 3 times. The average length of hospital stay was 36.2 ± 21.1 days, and the median was 33 days. Both fatalities were linked to *Aeromonas* infection, with severe sepsis and subsequent multiple organ dysfunction syndrome identified as the primary cause of death. Emergency amputations were performed in 2.70% (*n* = 3) of cases due to severe limb damage that precluded replantation upon admission, with the remaining 9.91% (*n* = 11) conducted due to progressive secondary infection.

**Fig. 1:**
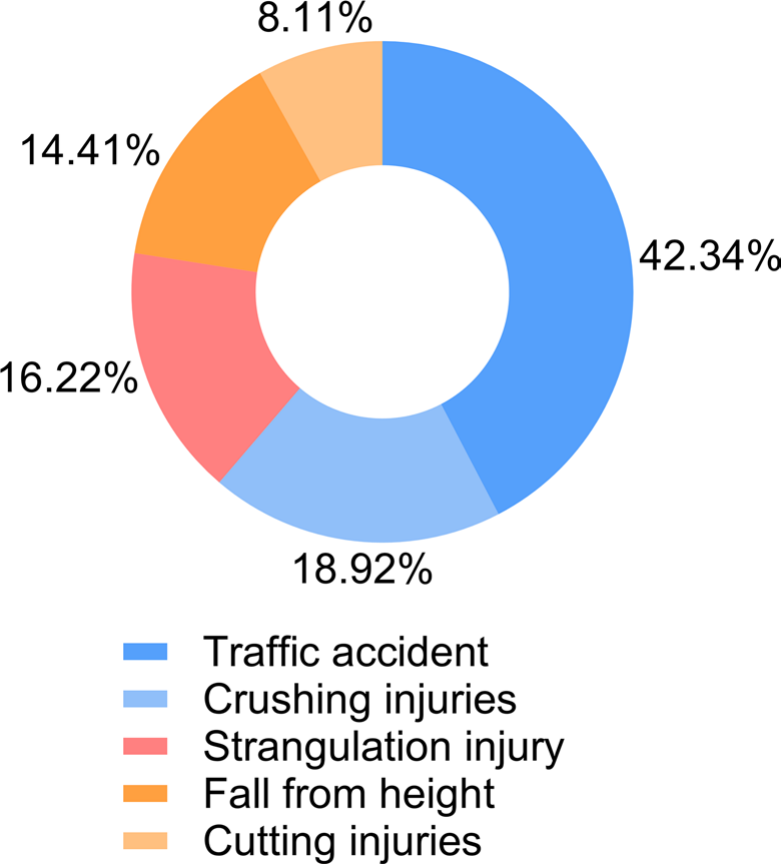
Injury cause distribution chart of the 111 patients

Among the 111 patients, the proportion of car accident injuries ranked highest (25 cases) in unfavourable prognosis, followed by strangulation injuries (10 cases) (Table 2). Statistically, the proportions of poor prognosis were relatively high for crushing injuries (18.92%) and traffic accidents (42.34%) (Fig. 1). This might be related to the fact that patients with these two injury mechanisms mostly suffered from high-energy injuries.

**Table 2:** The relationship between injury scenarios and prognosis

Variable	Parameter	Prognosis		*n*	X²	*p*-value
		Favorable	Unfavourable			
Season	spring	19(57.6%)	14(42.4%)	33	6.125	0.106
	summer	24(70.6%)	10(29.4%)	34		
	autumn	18(62.1%)	11(37.9%)	29		
	winter	5(33.3%)	10(66.7%)	15		
weather	sunny	28(65.1%)	15(34.9%)	43	2.188	0.335
	cloudy	15(65.2%)	8(34.8%)	23		
	rainy	23(51.1%)	22(48.9%)	45		
Injury cause	Traffic accident	22(46.9%)	25(53.2%)	47	14.314	0.006**
	Crushing injuries	14(66.7%)	7(33.3%)	21		
	Strangulation injury	8(44.4%)	10(55.6%)	18		
	Fall from height	15(93.8%)	1(6.2%)	16		
	Cutting injuries	7(77.8%)	2(22.2%)	9		
water contact	yes	1(50%)	1(50%)	2	2.168	0.141
	no	28(54.9%)	23(45.1%)	51		
	undetermined	-	-	58		

Note: ***, **, and * represent significance levels of 5%, 1% and 0.1% respectively

### Epidemiological investigation

Our hospital is home to the largest and most comprehensive orthopedic center in East China. We routinely admit and treat patients from Fujian Province and surrounding regions, performing over 20,000 orthopedic surgeries annually. To a certain extent, it can serve as a microcosm in the southeast coastal regions. From the frequency analysis, the weather with the highest incidence of *Aeromonas* infection and the highest unpromising prognosis (45 cases, 48.9%) both occurred on rainy days, indicating that rainy days may be a related factor for the prognosis of patients. Among the patients with a clearly documented exposure history (*n* = 53), only two had no history of water contact, while the vast majority (*n* = 51) did. Among these patients with a known exposure history, the ratio of favorable to poor prognosis was comparable, regardless of whether water exposure occurred. Yates’ corrected chi-square test yielded a *p*-value of 1.00, indicating no statistically significant association between prognosis and water exposure at the injury scene (Table 2).

### Relevant clinical information

Among patients with visible fractures, the probability of suboptimal clinical course was 51.4%, which was much higher than that in patients with invisible fractures (18.9%). The Fisher’s exact test yielded a statistically significant result (*p* < 0.01). It was found that patients with skin defects accounted for 67.7% of the poor prognostic outcomes. The *p*-value is 0.000***. Briefly, both skin defects and visible fractures were prognostically unfavourable.

Pearson’s chi-square test revealed a significant association between perioperative blood transfusion and prognosis (*p* < 0.01). Transfused patients constituted 48.9% (22/45) of the unfavorable prognosis group versus only 19.7% (13/66) of the favorable prognosis group (Table 3). The differences in haemoglobin and red blood cells postoperatively showed a decrease in more than 87% of the patients, which is one of the key indicators that need to be monitored after the operation (Table 4).

**Table 3:** The influence of clinical manifestations on prognosis

Variable	Index	Prognosis		*p*-value
		Favourable	Unfavourable	
Visible fracture	yes	36(48.6%)	38(51.4%)	0.001**
	no	30(81.1%)	7(18.9%)	
Defect of skin	yes	10(32.3%)	21(67.7%)	0.000***
	no	56(70.0%)	24(30.0%)	
Perioperative blood transfusion	yes	53(70.7%)	22(29.3%)	0.001**
	no	13(36.1%)	23(63.9%)	

Note: ***, **, and * represent significance levels of 5%, 1% and 0.1% respectively

Patients’ body temperature was monitored during treatment, including the initial heating temperature, maximum body temperature, and time from injury to first fever (in days). Among the variables, the regression coefficient of the highest body temperature was 0.799, and the *p*-value was 0.043*, indicating a positive correlation with adverse clinical outcome. This effect demonstrated statistical robustness within a 95% confidence interval ranging from 1.025 to 4.826 (Table 4).

**Table 4:** Regression analysis of binary variables and poor prognosis

Variable	Regression coefficient	Standard error	Wald	*p*-value	OR	95% confidence interval (OR)	
						Ceiling	Floors
Constant	-3.819	15.644	0.06	0.807	0.022	0	4.5489E + 11
Weather humidity	-0.012	0.026	0.214	0.644	0.988	0.938	1.04
Initial heating temperature	-0.704	0.48	2.15	0.143	0.494	0.193	1.267
Time of first fever	-0.003	0.076	0.001	0.973	0.997	0.859	1.158
Maximum body temperature	0.799	0.395	4.092	0.043*	2.224	1.025	4.826

Note: ***, **, and * represent significance levels of 5%, 1% and 0.1% respectively

### The drug sensitivity test

**Fig. 2:**
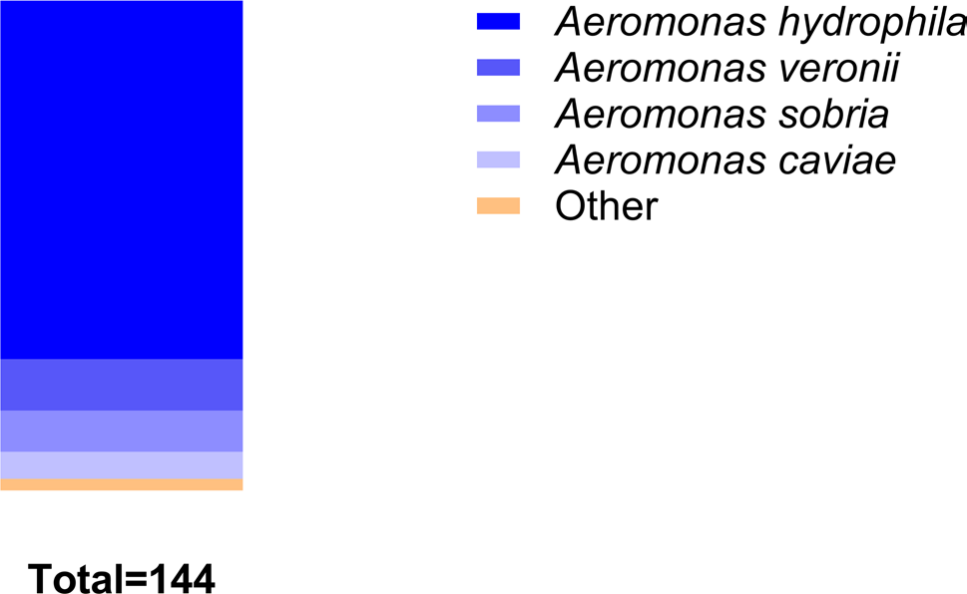
Proportion of different bacterial genera in *Aeromonas* infection

From 2018 to 2024, secretion cultures from 111 patients yielded 144 isolates, including 105 strains (72.9%) of *A. hydrophila*, 15 strains (10.4%) of *Aeromonas verona*, 12 strains (8.3%) of *Aeromonas sobria (A. sobria)*, 8 strains (5.6%) of *Aeromonas caviae (A. caviae)* and 4 other *Aeromonas* strains (2.8%) (Fig. 2). We defined *Aeromonas* strains with differing resistance profiles that emerged during treatment as distinct types. Consequently, the number of analyzed isolates exceeded the number of patients. Sequential sampling was conducted for this analysis. The specimens were all sourced from the secretions of traumatic wounds from the orthopedic ward.

**Fig. 3:**
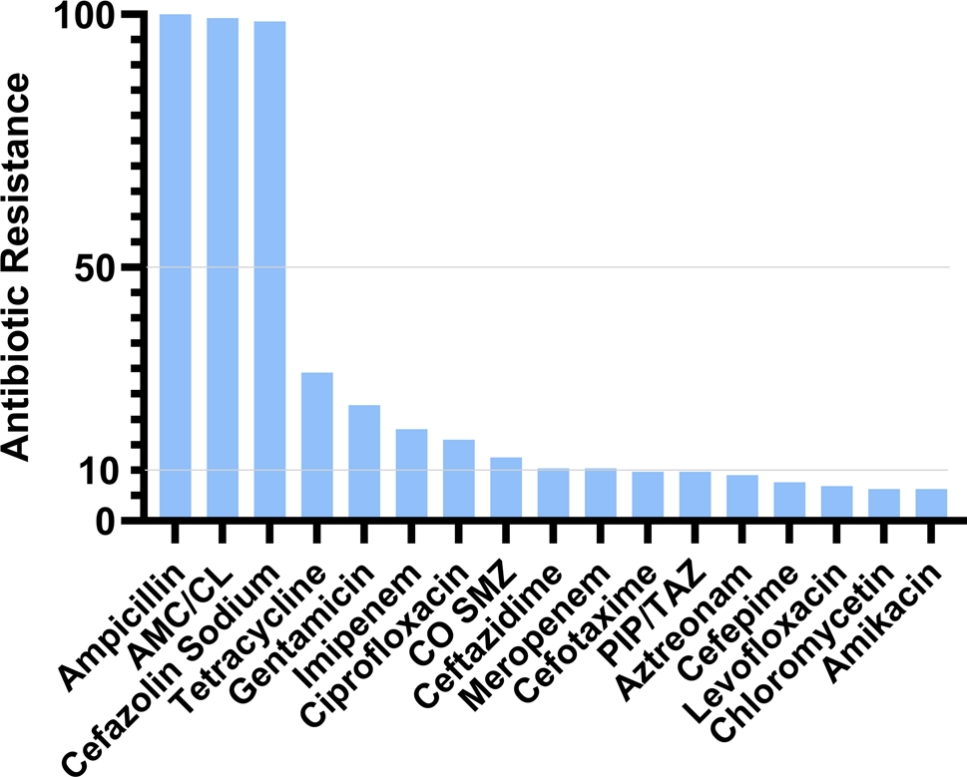
Drug susceptibility results of 144 *Aeromonas* strains

The drug sensitivity test results showed that *Aeromonas* extracted from the wound secretions were resistant to ampicillin, amoxicillin and clavulanate potassium (AMC/CL) and cefazolin sodium. The resistance rates to amikacin, chloromycetin, levofloxacin, cefepime, aztreonam and piperacillin sodium and tazobactam sodium (PIP/TAZ) were lower than 10.0%. Resistance to ceftazidime, compound sulfamethoxazole (CO SMZ), ciprofloxacin, meropenem, and imipenem was observed to be in the range of 10% to 20% (Fig. 3). The drug resistance rates of different species of *Aeromonas* vary. Generally, the drug resistance rate of *A. caviae* is relatively high (39.7%) among different genera, while that of *A. sobria* is relatively low (20.6%).

### Antibiotic usage

Prophylactic use of third-generation cephalosporins, aminoglycosides, or related dual antibiotic regimens (e.g., oxazolidinones combined with carbapenems) was associated with favorable outcomes. Empiric regimens including quinolones, third-generation cephalosporins with quinolones, aminoglycosides with glycopeptides, or oxazolidinones with carbapenems also showed acceptable therapeutic results. However, 95% confidence intervals indicated no statistically significant differences compared with the reference group (Table 5).

**Table 5:** The association between prophylactic use of antibiotics and prognosis

Groups	Mean Ridit Value	95% confidence interval	X²	*p*-value
Blank control	0.297	-0.029 ~ 0.624	14.059	0.230
Penicillins	0.503	0.406 ~ 0.600		
Quinolones	0.397	0.144 ~ 0.650		
Aminoglycosides	0.797	0.471 ~ 1.124		
First-generation cephalosporin	0.478	0.384 ~ 0.572		
Second-generation cephalosporin	0.497	0.244 ~ 0.750		
Third-generation cephalosporin	0.524	0.307 ~ 0.582		
Penicillins + Quinolones	0.297	-0.269 ~ 0.863		
Penicillins + Aminoglycosides	0.797	0.397 ~ 1.197		
Third-generation cephalosporins + Quinolones	0.797	0.471 ~ 1.124		
Third-generation cephalosporins + Aminoglycosides	0.797	0.231 ~ 1.363		

### Laboratory indexes

Laboratory tests from paired pre- and postoperative blood samples were compared. Through the analysis of laboratory indicators, it was concluded that 73.874% of the patients had decreased white blood cells and 56.757% of the patients presented with decreased platelets postoperatively. The proportions of patients with increased aspartate aminotransferase (AST) after the operation were 56.757%. Similar to alanine aminotransferase (ALT), the difference in AST also reflected the impact of the surgery on liver function. After the operation, 81.982% of the patients had decreased albumin levels, and 61.261% of the patients had decreased creatinine levels. A decline in serum creatinine levels often reflects improved renal perfusion and function, indicating effective treatment and a positive clinical trajectory. This observation is consistent with the finding that 66 out of the 111 patients (59%) had a favorable prognosis. The C-reactive protein (CRP) and procalcitonin (PCT) levels increased in 66.667% and 45.946% of the patients, respectively. As a sensitive indicator of bacterial infection, the increase in PCT may suggest the risk of infection after the operation. The erythrocyte sedimentation rate (ESR) of 63.964% of the patients increased after the operation, reflecting the existence of nonspecific inflammatory responses in the body after the operation (Table 6).

**Table 6:** Table of changes in postoperative and preoperative test indicators

Variable	Parameter	*n*	Percentage (%)	Cumulative percentage (%)
White blood cell difference	decrease	82	73.874	73.874
	increase	28	25.225	99.099
	invariant	1	0.901	100.000
Red blood cell difference	decrease	97	87.387	87.387
	increase	13	11.712	99.099
	invariant	1	0.901	100.000
Hemoglobin difference	decrease	97	87.387	87.387
	increase	12	10.811	98.198
	invariant	2	1.802	100.000
Platelet difference	decrease	63	56.757	56.757
	increase	47	42.342	99.099
	invariant	1	0.901	100.000
ALT difference	decrease	52	46.847	46.847
	increase	54	48.649	95.496
	invariant	5	4.505	100.000
AST difference	decrease	45	40.541	40.541
	increase	63	56.757	97.298
	invariant	3	2.703	100.000
CRP(mg/L) difference	decrease	20	18.018	18.018
	increase	74	66.667	84.685
	invariant	17	15.315	100.000
PCT(ng/ml) difference	decrease	12	10.811	10.811
	increase	51	45.946	56.757
	invariant	48	43.243	100.000
ESR(mm/h) difference	decrease	8	7.207	7.207
	increase	71	63.964	71.171
	invariant	32	28.829	100.000
Albumin (g/L) difference	decrease	91	81.982	81.982
	increase	18	16.216	98.198
	invariant	2	1.802	100.000
Creatinine difference	decrease	68	61.261	61.261
	increase	41	36.937	98.198
	invariant	2	1.802	100.000

### Likelihood ratio test for the overall regression model

The overall model validity was confirmed by a significant likelihood ratio test (*p* < 0.05), demonstrating meaningful inclusion of independent variables (Table 7).

**Table 7:** Likelihood ratio test for the binary logistic regression model

Model	-2 log likelihood	χ2	df	*p*-value	AIC	BIC
Intercept Only	146.706					
Final Model	112.928	33.778	3	0.000	120.928	131.656

Note: df (degrees of freedom); AIC (Akaike Information Criterion); BIC (Bayesian Information Criterion)

### Binary logit regression analysis

Significant indicators were first identified through univariate analysis. Using prognosis (favorable = 0, unfavorable = 1) as the dependent variable and variables including gender, BMI, age, perioperative blood transfusion, skin defect, visible fracture, postoperative changes in white blood cells, hemoglobin, red blood cells, platelets, ESR, albumin, ALT, AST, and creatinine as independent variables, a logistic regression model was constructed with stepwise selection. Following internal validation, the model retained three predictors: BMI, perioperative blood transfusion, and skin defect. The final regression equation was: ln(*p*/(1-*p*)) = -8.211 + 0.215*BMI + 0.566*perioperative blood transfusion + 0.536*skin defect.

BMI showed a significant positive association with poor prognosis (β = 0.215, z = 3.203, *p* = 0.001, OR = 1.240), indicating each unit increase in BMI raised the risk by 1.24 times. Similarly, both perioperative blood transfusion (β = 0.566, z = 2.556, *p* = 0.011, OR = 1.761) and skin defect (β = 0.536, z = 2.077, *p* = 0.038, OR = 1.710) were significant risk factors, increasing the likelihood of poor prognosis by 1.76 and 1.71 times, respectively. In summary, higher BMI, perioperative transfusion, and skin defect all significantly elevated the risk of poor prognosis, with OR values greater than 1 confirming them as risk factors (Table 8).

**Table 8:** Binary logit regression analysis of factors related to poor prognosis

Items	Regression Coefficient	Standard Error	z-value	Wald χ2	*p*-value	OR	95% confidence interval (OR)
Intercept	-8.211	1.840	-4.461	19.904	0.000	0.000	0.000 ~ 0.010
BMI	0.215	0.067	3.203	10.261	0.001**	1.240	1.087 ~ 1.415
Defect of skin	0.566	0.221	2.556	6.532	0.011*	1.761	1.141 ~ 2.717
Perioperative blood transfusion	0.536	0.258	2.077	4.313	0.038*	1.710	1.031 ~ 2.837

Note: ***, **, and * represent significance levels of 5%, 1% and 0.1% respectively

### Internal calibration

The Hosmer-Lemeshow test assessed model fit. The null hypothesis (that predicted values match observed values) was not rejected (χ²=8.813, *p* > 0.05), indicating adequate model fit (Table 9).

**Table 9:** Hosmer-Lemeshow goodness-of-fit test

χ2	df	*p*-value
8.813	8	0.358

### Binary logistic regression of prediction accuracy

Based on the confusion matrix, the model achieved a prediction accuracy of 90.740%, with misclassification rates of only 7.937% for class 0 and 11.111% for class 1 (Table 10).

**Table 10:** Binary logistic regression of prediction accuracy

		Predicted value		Prediction accuracy	Misclassification rate
		0	1		
Actual value	0	58	5	92.063%	7.937%
	1	5	40	88.889%	11.111%
Overall				90.740%	9.260%

### ROC curve analysis of logistic regression prognostic model

ROC curves were constructed for BMI, perioperative blood transfusion, and skin defect to evaluate their diagnostic value. The analysis revealed AUC values of 0.751 (95% CI: 65.77%-84.52%) for BMI, 0.819 (95% CI: 73.40%-90.36%) for perioperative blood transfusion, and 0.745 (95% CI: 64.70%-84.36%) for skin defect. These results indicate that all three variables demonstrate substantial diagnostic value for the condition (Table 11).

**Table 11:** ROC AUC results of the prediction model

Risk factor	AUC	Standard error	*p*-value	95% confidence interval
BMI	0.751	0.048	0.000***	0.658 ~ 0.845
Skin defect	0.745	0.050	0.000***	0.647 ~ 0.844
Perioperative blood transfusion	0.819	0.043	0.000***	0.734 ~ 0.904

Note: ***, **, and * represent significance levels of 5%, 1% and 0.1% respectively

**Fig. 4:**
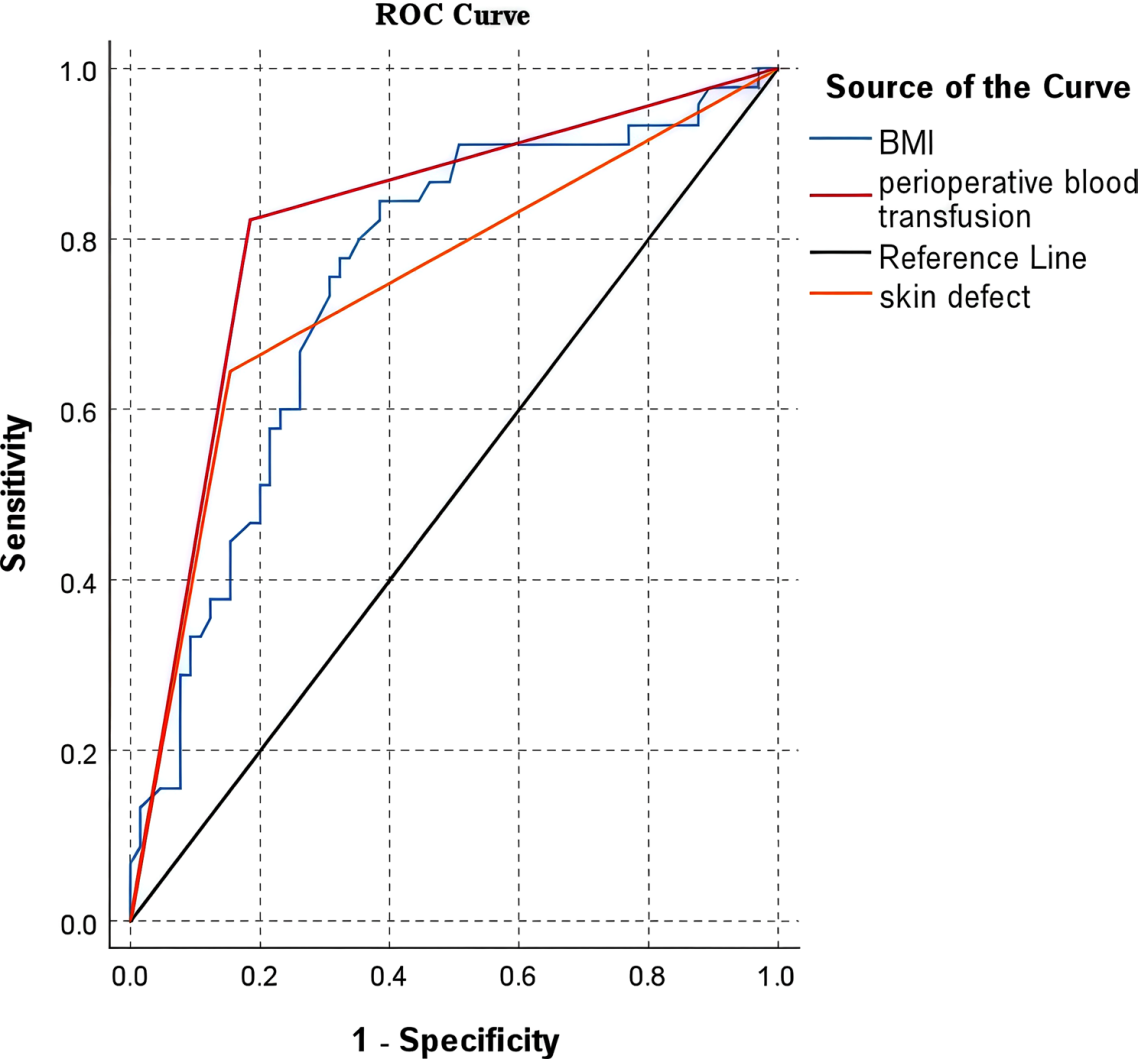
ROC curve analysis of logistic regression prognostic model

BMI showed an AUC of 0.751, indicating substantial diagnostic value, with an optimal cutoff of 0.460 (sensitivity 0.844, specificity 0.615). Based on ROC analysis, the optimal predictive cut-off value was 23.7 kg/m², indicating that patients with BMI > 23.7 kg/m² have a significantly increased risk of poor prognosis. Perioperative blood transfusion demonstrated an AUC of 0.819 and an optimal cutoff of 0.638 (sensitivity 0.822, specificity 0.815). Skin defect yielded an AUC of 0.745 with an optimal cutoff of 0.491 (sensitivity 0.644, specificity 0.846) (Table 12; Fig. 4).

**Table 12:** Optimal cut-off values from ROC analysis

Risk factor	AUC	Optimal Cut-off Value	Sensitivity	Specificity	Cut-off
BMI	0.751	0.460	0.844	0.615	23.700
Skin defect	0.745	0.491	0.644	0.846	0.000
Perioperative blood transfusion	0.819	0.638	0.822	0.815	0.000

## Discussion

Multiple clinical studies have established that traumatic *Aeromonas* infections in the extremities substantially elevate the risk of NF, with an acute mortality rate of approximately 30% within two weeks of onset. This risk is particularly exacerbated in patients with comorbid conditions such as concurrent polymicrobial infections, liver cirrhosis, or malignant tumours [15–17]. In the early stage of *Aeromonas* NF, patients frequently presented with hemorrhagic bullae and skin necrosis. Surgical exploration revealed superficial venous embolism in the fascia layer [18, 19]. In the 7-year follow-up of patients with Gustilo IIIB open tibial fractures accompanied by *Aeromonas* infection, Tsunoda T et al. emphasised that thorough debridement and appropriate antibiotic treatment were the keys to successful treatment [20].

Existing studies generally indicate that *Aeromonas* infections exhibit seasonal characteristics, occurring more frequently during the warmer summer and autumn months [10]. However, this study found that although there is a seasonal clustering of infections (predominantly in spring and summer), no statistically significant association was observed between prognosis and factors such as the season, weather, humidity, or water exposure at the time of injury.

While previous studies have suggested associations between demographic factors like age and gender with postoperative outcomes [21–23], these were not independent predictors in our final multivariate model. In contrast, elevated BMI was identified as an independent risk factor for poor prognosis in traumatic *Aeromonas* infection (OR = 1.240, *p* = 0.001), with each unit increase raising the risk of unfavorable outcomes by 1.24-fold. Notably, the optimal predictive cutoff was 23.7 kg/m²—slightly below the Chinese overweight threshold (BMI ≥ 24 kg/m²) yet clinically significant. This lower threshold implies that patients in the high-normal weight range already face substantially increased risk, potentially due to metabolic dysregulation, chronic inflammation, and impaired tissue perfusion associated with higher BMI, which collectively compromise host defense and tissue repair [24, 25]. Consequently, trauma patients with BMI approaching or exceeding 23.7 kg/m² should be considered high-risk and warrant heightened vigilance and more aggressive management.

In terms of clinical characteristics, perioperative blood transfusion (OR = 1.761, *p* = 0.011) and skin defects (OR = 1.710, *p* = 0.038) were identified as independent predictors of poor prognosis, with their diagnostic value further confirmed by ROC analysis (AUC: 0.819 and 0.745, respectively). Skin defects—indicative of severe trauma and impaired tissue repair—often necessitate complex reconstruction (e.g., grafting or flaps), prolonging recovery and elevating complication risks. Consistent with clinical observations, such defects significantly increase the likelihood of poor outcomes, including amputation, particularly in the context of high-energy injuries or subsequent infection [26]. These findings underscore the need for heightened vigilance in managing patients with significant skin loss. Perioperative blood transfusion reflects greater baseline severity and independently contributes to poor outcomes through multiple pathways: immunomodulation (simultaneous immunosuppression and pro-inflammation), storage lesion-induced microcirculatory dysfunction, and the inherent risks of severe anemia [27–29]. These mechanisms collectively increase infection risk, complicate recovery, and elevate mortality, underscoring the need for vigilant hemodynamic management in high-risk patients. Although visible fractures showed significance in univariate analysis, they were not retained in the multivariate model, suggesting their effect may be mediated through more direct indicators of soft tissue damage such as skin defects.

Notably, several laboratory parameters—including albumin, hemoglobin, liver function, etc—which have been implicated as prognostic markers in previous studies [30–32], did not maintain independent prognostic value in our final multivariate model. This indicates that while these markers may reflect physiological status, their predictive power is superseded by the retained clinical factors in the context of *Aeromonas*-infected trauma patients.

The antimicrobial resistance profile observed in our study confirms established patterns of *Aeromonas* susceptibility. Our findings support the use of third-generation cephalosporins combined with quinolones or aminoglycosides for high-risk patients, while discouraging first-generation cephalosporins as first-line therapy [33]. Sun et al. found that all 16 cases with limited therapeutic response had inappropriate empirical antibiotic treatment [34], underscoring the importance of appropriate initial antimicrobial selection. However, it is crucial to recognize that standard susceptibility testing cannot fully replicate the complex in vivo microenvironment, including biofilm formation and limited tissue penetration. The possibility of polymicrobial infections or sampling bias must also be considered.

The logistic regression model developed in this study provides a quantitative framework for postoperative prognosis assessment, serving as an exploratory effort that lays the groundwork for further refinement of disease models. In conclusion, the model-identified risk factors (BMI > 23.7, perioperative blood transfusion, skin defects) should guide initial empiric therapy toward *Aeromonas* coverage using susceptibility-confirmed agents. According to established guidelines, the prophylactic antibiotic regimen for open fractures should be based on the Gustilo-Anderson classification [35]. First-generation cephalosporins serve as the foundational therapy. For Type III fractures (IIIA, IIIB, IIIC)—characterized by extensive soft tissue damage, contamination, or vascular compromise—expanded Gram-negative coverage is essential, particularly targeting *P. aeruginosa*. The recommended regimen combines a first-generation cephalosporin (clindamycin for β-lactam allergic patients) with an aminoglycoside (gentamicin) or fluoroquinolones as alternatives. Third-generation cephalosporins (e.g., ceftriaxone) or PIP/TAZ may also be considered [36–38].

Notably, *Aeromonas* species demonstrate significant resistance to first-generation cephalosporins but remain susceptible to third-generation cephalosporins and aminoglycosides. While Gustilo Type III protocols inherently cover *Aeromonas* through broad-spectrum antibiotics, Type I and II regimens—lacking routine Gram-negative coverage—may fail to address *Aeromonas* infections adequately. Our study extends these guidelines by proposing that even in non-fracture limb injuries with skin defects, BMI > 23.7 kg/m^2^, or perioperative transfusion, prophylactic use of third-generation cephalosporins (e.g., ceftazidime), aminoglycosides (e.g., amikacin), or combination therapy should be considered. First-generation cephalosporins are not recommended as first-line agents in these scenarios. Similarly, Gustilo Type I/II fractures with these risk factors warrant *Aeromonas* coverage. Enhanced perioperative monitoring and tailored management are crucial to identify febrile reactions, chills, or invasive infections—particularly necrotizing fasciitis, myositis, and osteomyelitis—in immunocompromised patients.

## Conclusions

*Aeromonas* infection progresses insidiously and rapidly, with a lack of corresponding standards for judging clinical prognosis. This retrospective study analysed the clinical characteristics and epidemiological data of 111 patients with traumatic *Aeromonas* infection. The results showed that the infection predominantly affected middle-aged individuals (mean age: 49.9 years), with prolonged hospital stays (mean: 36.2 days) and multiple surgical interventions (mean: 2.7 procedures). Substantial rates of severe adverse outcomes were observed, with amputation at 12.61% and mortality at 1.80%. Antimicrobial susceptibility testing revealed high resistance rates to common antibiotics such as ampicillin, but maintained sensitivity to amikacin and levofloxacin. Through univariate and logistic regression analysis, three independent predictors of poor prognosis were identified, including BMI > 23.7 kg/m^2^, skin defects and perioperative blood transfusion. For high-risk trauma patients with these factors, our model recommends prophylactic third-generation cephalosporins (e.g., ceftazidime), aminoglycosides (e.g., amikacin), or dual therapy instead of first-line cephalosporins. Enhanced perioperative monitoring is crucial to detect febrile reactions, chills, and invasive infections—particularly necrotizing fasciitis, myositis, and osteomyelitis—especially in immunocompromised patients.

### Limitations

This study has several limitations that should be considered. First, as a single-center retrospective analysis with a limited sample size, the generalizability of our regression model requires validation in independent populations. Second, the absence of a control group (e.g., trauma patients without *Aeromonas* infection) limits the ability to specifically attribute poor outcomes to *Aeromonas*. Additionally, the retrospective design may introduce information bias, as some clinical data were incompletely documented. Furthermore, since cases of mixed infections were excluded, the predictive performance of this model in such contexts remains unclear and warrants further investigation. Despite these limitations, the model provides valuable prognostic insights for monomicrobial *Aeromonas* infections, though clinicians should also consider patients’ underlying conditions in practice.

## Data Availability

The datasets used and/or analysed during the current study are available from the corresponding author on reasonable request. Please contact the corresponding author, Dr Lin. Administrative permission was received from Fuzhou Second General Hospital (No. 47, Shangteng Road, Cangshan District, Fuzhou, China) to access the medical records.

## Abbreviations

AIC: Akaike Information Criterion
ALT: Alanine Aminotransferase
AMC/CL: Amoxicillin and Clavulanate Potassium
AST: Aspartate Aminotransferase
AUC: Area Under the ROC Curve
BIC: Bayesian Information Criterion
BMI: Body Mass Index
CLSI: Clinical and Laboratory Standards Institute
CO SMZ: Compound Sulfamethoxazole
CRP: C-reactive Protein
ESR: Erythrocyte Sedimentation Rate
NF: Necrotizing Fasciitis
PCT: Procalcitonin
PIP/TAZ: Piperacillin Sodium and Tazobactam Sodium
SSIs: Surgical site infections

## Microbiological nomenclature

*A. caviae*: *Aeromonas caviae*
*A. hydrophila*: *Aeromonas hydrophila*
*A. sobria*: *Aeromonas sobria*
*P. aeruginosa*: *Pseudomonas aeruginosa*
